# Automatic Frequent Measurements of Plasma Sodium, Potassium, Total Carbon Dioxide, Urea Nitrogen, and Other Values During Hemodialysis

**DOI:** 10.7759/cureus.113660

**Published:** 2026-07-30

**Authors:** Susie Q Lew, Todd S Ing, Ramin Sam, Susie N Pham, Deepak Malhotra, Antonios H Tzamaloukas

**Affiliations:** 1 Department of Medicine, The George Washington University School of Medicine and Health Sciences, Washington, D.C., USA; 2 Department of Medicine, Stritch School of Medicine, Loyola University Chicago, Maywood, USA; 3 Division of Nephrology, University of California in San Francisco, San Francisco, USA; 4 Department of Medicine, University of Toledo School of Medicine, Toledo, USA; 5 Department of Internal Medicine, University of New Mexico School of Medicine, Albuquerque, USA

**Keywords:** blood gas measurement, dialysis machine, electrolyte measurements, hemodialysis, point-of-care blood tests

## Abstract

Customizing acute and chronic hemodialysis treatments by tailoring the prescription can occur by knowing the patient’s electrolyte and acid-base status at any given moment. Point-of-care (POC) testing using whole blood samples can provide laboratory results of sodium, potassium, chloride, bicarbonate, glucose, calcium, phosphorus, blood urea nitrogen, creatinine, pH, partial pressure of carbon dioxide, oxygen, and lactate. The impact of dialysis on these values will impact patient outcomes and quality of life. Besides knowing the laboratory results, one can calculate the anion gap and osmolar gap to treat patients with disorders such as diabetic ketoacidosis and alcohol poisoning, respectively. We describe a new technology for obtaining blood samples from the hemodialysis arterial line and using a POC device installed in the dialysis machine to perform electrolyte and blood gas measurements, and inform the clinician promptly of possible prescription adjustments to meet the needs of the patient requiring acute or chronic hemodialysis. Besides the obvious benefits, we highlight some pitfalls associated with using whole blood versus serum samples, and arterial versus venous blood samples.

## Introduction

It is often clinically necessary to promptly know the results of a given dose of hemodialysis on plasma electrolytes, urea nitrogen, and other levels during the course of a dialysis treatment [1]. Based on this information, changes in dialysis fluid composition, dialyzer blood flow rate, dialysis fluid flow rate, duration of dialysis, and other treatment parameters can be made readily if necessary. At present, in dialysis units, if one wishes to know such laboratory results, one needs to draw blood from the arterial blood side and deliver the aspirated blood sample to a clinical laboratory. The latter determines the requested parameters and then reports the results back to the responsible health care personnel. All these steps take time, often affecting prompt and critical decision-making.

This unnecessary obstacle can be easily circumvented by obtaining a blood sample from a port originating from the arterial blood tubing and delivering this blood sample to a device incorporated in the dialysis machine that measures the requested values throughout the course of a dialysis session. This process will be described below.

## Technical report

Knowing certain laboratory values in patients with acute kidney injury will dictate the subsequent dialysis treatment(s). Beginning January 1, 2017, Medicare reimburses dialysis services for patients with acute kidney injury in end-stage renal disease (ESRD) facilities [2]. Any blood samples obtained at an outpatient dialysis unit must be sent to a national or a dialysis-affiliated laboratory service since the dialysis unit does not have the capability to conduct such analysis. The results will appear days later. Similarly, any blood samples obtained at an acute dialysis facility will be sent to the hospital laboratory, with results available in a few hours. The delay in knowing certain laboratory results impacts and delays applying the appropriate dialysis prescription.

We therefore propose a method to obtain certain laboratory values at the point of care (POC), whether it be in the chronic or acute dialysis unit, intensive care unit, or emergency department. The blood sample can be obtained at the start or during the dialysis treatment. The POC device will be incorporated into the dialysis machine. Blood sampling will be automated. The results will be displayed on the dialysis machine and incorporated into the electronic medical record with the appropriate connection.

**Figure 1:**
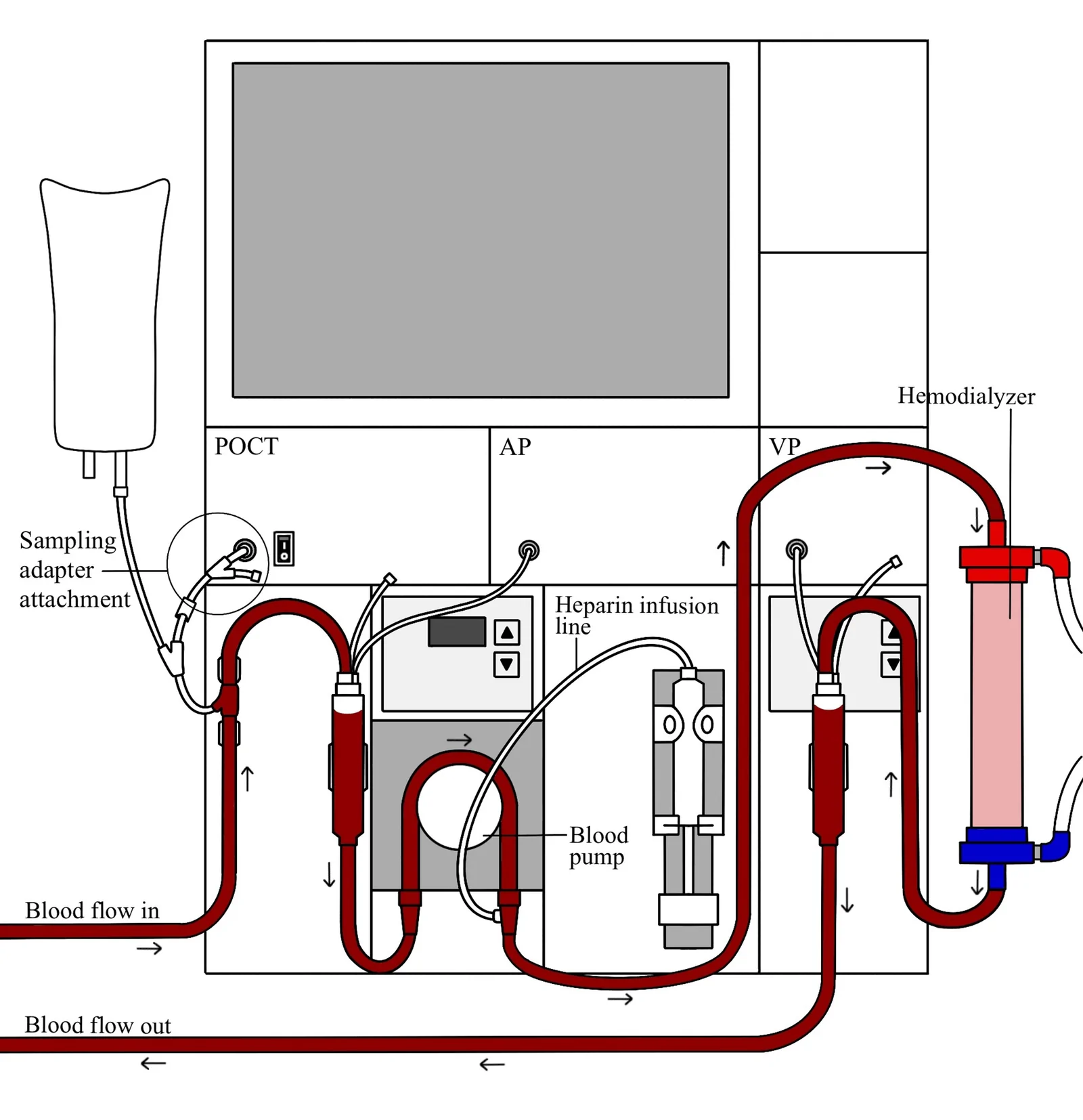
A schematic image of a hemodialysis machine with a point-of-care testing module that can access blood for electrolyte or blood gas analysis during a dialysis treatment POCT: point-of-care testing (POCT module performs electrolyte or blood gas analysis during a dialysis treatment.); AP: arterial pressure (AP module measures pressure in the arterial line to ensure that air does not enter the dialyzer. In hemodialysis, the arterial line contains blood leaving the patient to enter the dialyzer. This blood may be a mixture of arterial and venous samples from an arteriovenous fistula or arteriovenous graft, or a venous sample from a double-lumen venous dialysis catheter.); VP: venous pressure (VP module measures pressure in the venous line to ensure that air does not get delivered to the patient. In hemodialysis, the venous line contains blood returning to the patient.) Heparin prevents blood from clotting when added to the blood via the heparin infusion line. A special adapter added to the saline infusion line will allow frequent automatic blood sampling for electrolyte or blood gas analysis from the arterial line. This figure was created using Procreate (Savage Interactive Pty Ltd, Hobart, Australia).

Figure 1 shows a proposed scheme for POC measurements during a hemodialysis session. The scheme consists of the following four technical steps. 

Blood sampling. Blood must be obtained or sampled either continuously or at desired intervals. A typical arterial tubing for conventional hemodialysis has an arterial split septum injection site, saline access site (part of the saline priming set), arterial “pigtail” access site, and monitor line (which will connect to the transducer) with both as an attachment to the arterial chamber and a heparin access site. An ideal sampling site would have continuous blood flow and be short in length to avoid sampling stagnant blood. We envision a creative engineer designing and fabricating a blood sampling device if the currently available arterial tubing is to be used. A possible first device would repetitively aspirate a defined quantity of blood via a sterile needle inserted into the arterial split septum injection site. Another possible option would be a device that could advance a sterile siphon via the arterial “pigtail” into the arterial chamber for a defined blood sample and then retract until needed again. Alternatively, manufacturers of dialysis tubing can redesign the arterial tubing line to have a new blood sampling port with the specifications listed previously.

POC device installation. The second requirement would be to install a POC testing device as a module in the dialysis machine. This POC testing device would perform a panel of tests pertinent to dialysis (i.e., sodium, potassium, calcium, blood urea nitrogen, creatinine, or blood gas) and provide results in real time. Currently, POC tests occur in a wide range of locations including the home, health care practitioner’s office, emergency department, ambulances, military, radiology department, cruise ship, space shuttle, and intensive care unit using a hand-held or countertop device [3,4].

Activation of the blood drawing mechanism. A mechanism to activate this process manually or at a pre-set time interval via a timer should be placed in this system.

Presentation of the results of the measurements. The results of the test need to be displayed on the dialysis machine screen or as a printout. The results can be included in the patient’s electronic medical record with the appropriate connection.

## Discussion

Several clinical conditions treated in intensive care units by hemodialysis may require POC testing during a dialysis session. We will use hemodialysis for a few categories of acute conditions to illustrate the POC testing methods and the measurement devices needed for these treatments. POC testing is ideal for dialyzing patients in an intensive care unit setting with (a) complicated electrolyte disorders [1,5-8], (b) acid-base disorders [9-11], (c) combined severe dysnatremias and acid-base disorders [12], or (d) those with severe illness with or without renal failure, for example, patients with life-threatening intoxications by dialyzable substances [13], or monitoring response of septic shock to antibiotics by following lactate levels. Hemodialysis for severe intoxications by ethanol [14,15] or methanol [16,17] represents an example of this last category of conditions best monitored by POC.

POC monitoring of the correction of electrolyte abnormalities by hemodialysis requires devices measuring the concentrations of sodium, potassium, chloride, and total carbon dioxide in serum or plasma. The autoanalyzers currently used in the main hospital laboratories to measure these concentrations include potentiometry with pre-dilution of the serum sample (indirect potentiometry) or without pre-dilution of this sample (direct potentiometry). Indirect potentiometry carries the risk of pseudohyponatremia when measuring sodium concentration in serum or plasma with high values of solid content secondary to hyperproteinemia or hyperlipidemia [18]. For this reason, use of a device using direct potentiometry for POC measurements is preferable. Other POC measurements that can be used for the correction of electrolyte abnormalities, acid-base disorders, or certain intoxications include the concentrations in serum or plasma of glucose, urea, and osmolality.

The POC device which is essential for correction of acid-base abnormalities by hemodialysis is a blood gas apparatus. Blood gas apparatuses also measure plasma sodium concentration by direct potentiometry using whole blood. The serum anion gap, that is the difference between serum sodium, or sodium plus potassium, and chloride plus bicarbonate, or total carbon dioxide, is one other parameter which, when estimated by POC, can be useful during correction of endogenous metabolic acidosis by hemodialysis. Lactic acidosis with plasma lactate levels changes dictated by continuous production and removal during the course of a hemodialysis session is an example of the use of the anion gap instead of measuring serum lactate levels, which may not always be available. POC measurement of the concentrations of sodium and bicarbonate in the dialysis fluid may be needed after each change of the composition of this fluid when treating combined dysnatremias and acid-base disorders by hemodialysis [12].

The measurement of the concentrations of various alcohols is also not universally available. Absence of POC testing may cause either premature termination or unnecessary extension of a hemodialysis treatment for alcohol intoxication [19]. The osmolar gap, that is the difference between plasma osmolality and osmolarity computed as the sum of plasma sodium times two plus plasma glucose plus plasma urea, where glucose and urea concentrations are in mmol/L [20], is a reasonable substitution for alcohol concentration in this case [21]. A POC device measuring osmolality by a colligative property, for example, by determination of the freezing point of the solution tested, will be necessary for the monitoring of the osmolar gap, along with the POC measurements of sodium, glucose, and urea concentrations. 

One needs to be aware of factors that may provide inaccurate results. For example, discrepancies exist in reported electrolyte concentrations, including the concentration of sodium and potassium, between POC apparatuses, which use whole blood for their measurements, and central laboratories, which use serum for their measurements [22]. Another example would be the impact of recirculation associated with proximal arterio-venous access stenosis or with the use of a double lumen dialysis catheter, where uncleansed blood contaminates cleansed blood. Thus, the POC results reflect the dialysis fluid concentrations of electrolytes rather than the concentrations in a peripheral venous sample. In addition, one should be mindful of whether a mixture of arterial and venous blood (from an arterio-venous fistula or graft) or venous blood (from a double lumen venous catheter) is sampled. This is particularly important when interpreting the partial pressures of oxygen and carbon dioxide, and the pH.

Although there will be upfront costs associated with designing, testing, and manufacturing the POC module and adapter to the arterial blood line, the long-term cost savings can be substantial. There will be cost savings associated with not having to obtain blood samples, transport the sample to the laboratory, and perform the requested blood tests. As a result of having the result within minutes of sampling the blood, the dialysis treatment can be optimized with respect to dialysis fluid electrolyte concentration, and treatment time and frequency. This can be further translated into cost of dialysis staff time, especially overtime, and the number of treatments a patient will need for a particular diagnosis. Physician rounding time will be more efficient, and the encounter will be more effective with up-to-the-minute electrolyte and acid/base data.

Additional steps to assess technical feasibility, safety considerations, device validation, and regulatory requirements will need to be proposed and tested prior to clinical implementation. The POC technology already exists and can be inserted in the dialysis machine as a module. The results can be programmed to be displayed on the screen and exported to an electronic health record. An adaptor will need to be designed, manufactured, and tested to ensure a safe and effective method of obtaining a blood sample from the dialysis circuit.

Overall, this concept of obtaining real-time blood gas and electrolyte results during a dialysis treatment will allow clinicians to tailor the dialysis prescription specifically for the patients’ needs to improve outcomes and quality of life. Could it be that personalized medicine is finally reaching the dialysis population?

## Conclusions

The use of POC technology during dialysis allows for prompt assay of blood for electrolytes and blood gas. We propose a schema to automate the process of obtaining blood samples from the arterial line and delivering the sample to a POC device module installed in the dialysis machine, where a panel of tests pertinent to dialysis can be performed. The blood test results can be used to change the dose of dialysis or the dialysis fluid composition. Some examples include the treatment of hyponatremia, alcohol intoxication, hyperkalemia, and lactic acidosis. When interpreting the results, the clinician should be aware that the whole blood sample will result in a plasma result and that the blood sample will be a mixture of arterial and venous if obtained from an arterio-venous access, and venous if obtained from a central venous catheter. The use of POC technology will allow clinicians to personalize the dialysis prescription to improve patient outcomes and quality of life.
